# Triglyceride-glucose-based indices in relation to vitamin D concentrations among adults with metabolic syndrome

**DOI:** 10.1371/journal.pone.0349683

**Published:** 2026-06-01

**Authors:** Raju Rana, Purnima Adhikari, Ullas Kamath, B. Ananthakrishna Shastri, Shashikiran Umakanth, G. Arun Maiya, Raghavendra Rao S, Vani Lakshmi R, Shobha U. Kamath

**Affiliations:** 1 Department of Biochemistry, Kasturba Medical College, Manipal Academy of Higher Education, Manipal, Karnataka, India; 2 Department of Anatomy, Kasturba Medical College, Manipal Academy of Higher Education, Manipal, Karnataka, India; 3 Department of Biochemistry, Melaka Manipal Medical College, Manipal Academy of Higher Education, Manipal, Karnataka, India; 4 Department of Medicine, Kasturba Medical College, Manipal Academy of Higher Education, Manipal, Karnataka, India; 5 Department of Medicine, Melaka Manipal Medical College, Manipal Academy of Higher Education, Manipal, Karnataka, India; 6 Department of Physiotherapy, Manipal College of Health Professions, Manipal Academy of Higher Education, Manipal, Karnataka, India; 7 Centre for Digital Health, Applied Research and Technology, Prasanna School of Public Health, Manipal Academy of Higher Education, Manipal, Karnataka, India; Tehran University of Medical Sciences, IRAN, ISLAMIC REPUBLIC OF

## Abstract

**Background:**

The concurrent global epidemics of 25-hydroxyvitamin D (25[OH]D) deficiency and metabolic syndrome (MetS) pose substantial public health challenges. This study investigated the association of the triglyceride-glucose (TyG) related indices with serum 25(OH)D concentration in MetS patients.

**Methods:**

In this study, we have recruited 1,297 participants (869 men, 428 women) with MetS who underwent regular health checkups between 2021 and 2023. Participants’ demographic, laboratory, and clinical parameters were retrieved, and data were divided into three groups based on the TyG index. Parameters were compared across the TyG index tertiles. Correlation analysis was used to find the association of the TyG index and related indices with 25(OH)D.

**Results:**

The TyG-body mass index (TyG-BMI) demonstrated a statistically significant but weak negative correlation with serum 25(OH)D levels (r = −0.09, P < 0.001), whereas no significant association was observed between the standard TyG index and 25(OH)D (r = 0.007; P = 0.81). Comparative analysis among tertiles revealed significant differences in lipid profiles and glucose parameters (P < 0.001). Males demonstrated significantly higher TyG index values (P < 0.001), while females showed significantly elevated TyG-BMI (P < 0.001). Serum 25(OH)D concentrations were significantly higher in males compared to females (19.2[8.87] vs 16.6[9.50] ng/mL, P < 0.001). Only 92 (7%) of our study population had a normal level of 25(OH)D (≥30 ng/mL).

**Conclusion:**

The association between TyG-BMI and vitamin D is weak and of limited clinical relevance. Further prospective studies should be conducted to evaluate the association of these indices with 25(OH)D.

## Introduction

Metabolic syndrome (MetS) is a cluster of conditions comprising hypertension, hyperglycemia, central obesity, and dyslipidemia, which are considered risk factors for the future development of type 2 diabetes mellitus (T2DM), cardiovascular disease (CVD), and certain cancers. The global prevalence of MetS is rising (12.5% to 31.4%), depending on the definition used, and is considered one of the major contributors to non-communicable disease mortality in developing countries [1].

25-hydroxyvitamin D (25[OH]D) deficiency (VDD) has arisen as a critical global health challenge, with increasing prevalence rates observed across diverse populations worldwide [2], particularly in South Asian countries like India [3]. Paradoxically, despite abundant sunlight, factors such as increased urbanization, reduced outdoor activities, and darker skin pigmentation aid in widespread VDD in the Indian community [3–5]. Emerging evidence has expanded our understanding of 25(OH)D’s role beyond calcium and phosphate regulation, linking its deficiency to increased risks of mortality, diabetes, thyroid dysfunction, obesity, cancer, and vascular disease [6,7].

Studies have shown inadequate 25(OH)D levels correlate with MetS [8, 9]. Furthermore, a comprehensive systematic review and meta-analysis of randomized controlled trials of T2DM patients revealed a moderate enhancement in insulin sensitivity following 25(OH)D supplementation interventions [10,11]. However, a recent meta-analysis indicates inadequate evidence supporting the advantages of taking 25(OH)D on MetS and its elements among obese and diabetic patients, highlighting the complexity of this relationship [12]. Similarly, a randomized controlled trial in prediabetes shows no effects of 25(OH)D supplements on insulin resistance, emphasizing the inconsistent conclusion about the interplay between 25(OH)D and insulin resistance [13].

However, due to its cost, 25(OH)D is not routinely tested in many hospitals. Further, studies have shown that most 25(OH)D test requests are unnecessary [14,15]. Therefore, it is important to develop a cost-effective screening tool for the detection of patients at risk of VDD. This could help relieve unnecessary financial burdens prior to sending for the test.

The Triglyceride-Glucose (TyG) index, derived from fasting plasma glucose (FPG) and triglyceride concentration, has gained attention as a reliable and cost-effective surrogate marker of insulin resistance [16]. A substantial body of literature has supported the concurrence of the TyG index and standard insulin resistance measurements, such as homeostatic measurement of insulin resistance (HOMA-IR) and hyperglycemic clamp [17,18]. Studies have elucidated its role as a marker for CVD deaths in the general population, mortality in MetS, and renal impairment in hypertensive patients [7,19–22]. Similarly, TyG-body mass index (TyG-BMI) has also shown a significant association with the standard measure of insulin resistance [23].

Despite growing evidence linking insulin resistance indices to metabolic complications, the relationship between triglyceride-glucose indices and 25(OH)D status in MetS remains poorly characterized. Therefore, this study aimed to conduct a retrospective cross-sectional analysis in patients with MetS to examine the associations between TyG and TyG-BMI indices with serum 25(OH)D concentrations, providing valuable insights for clinical practice.

## Materials and methods

### Study design and setting

**Fig 1:**
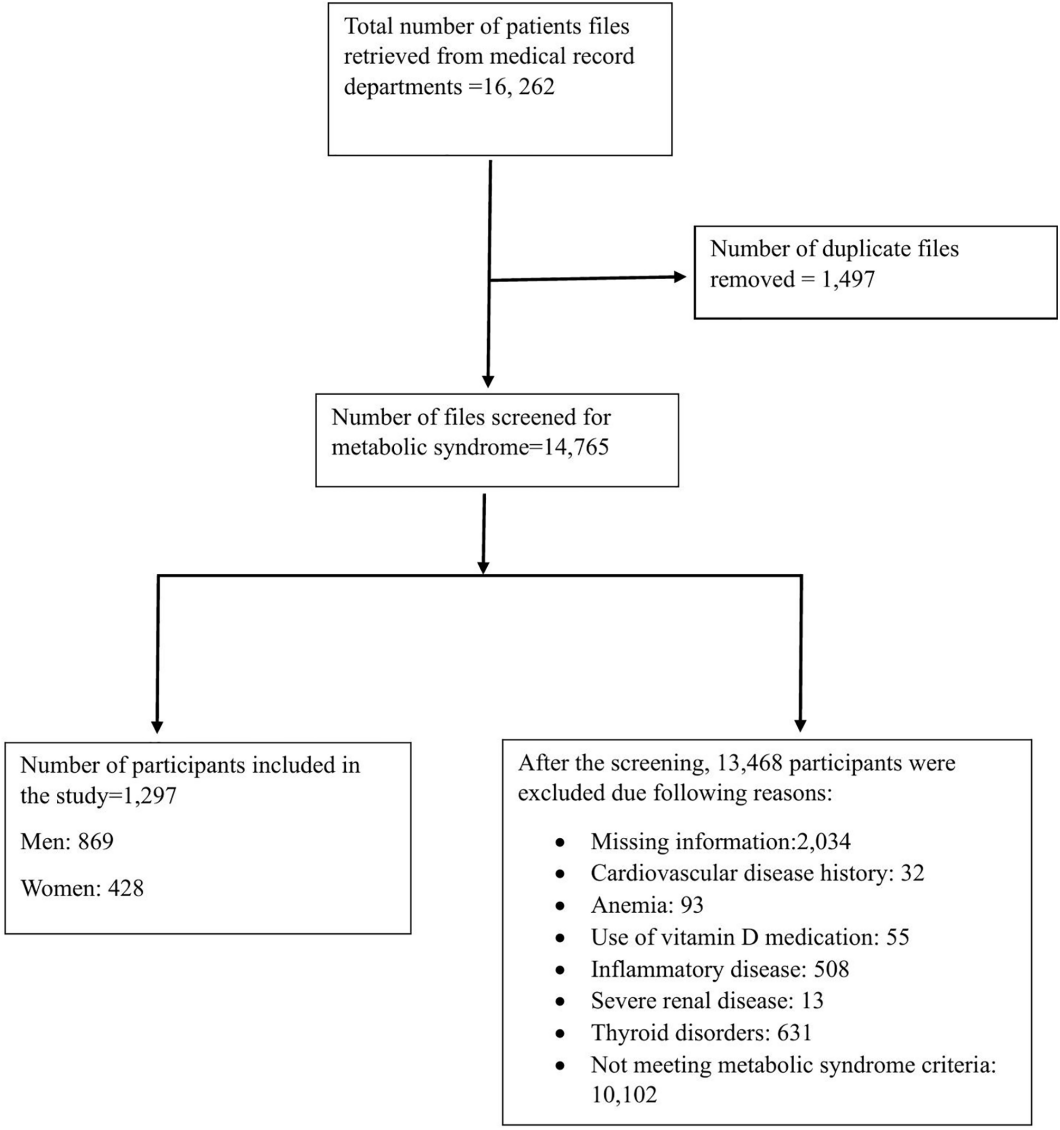
Study participants’ selection flowchart.

This retrospective cross-sectional study investigated the TyG index and TyG-BMI association with 25(OH)D in patients with MetS. Initially, 16,262 patients whose medical records were screened, 1,297 (869 men, 428 women) met the diagnostic criteria for MetS and were evaluated (Fig 1).‌‌

The TyG index values were stratified into three tertiles: tertile 1 (<9.16), tertile 2 (9.16–9.53), and tertile 3 (≥9.54). 25(OH)D, lipid profile, FPG, urea, creatinine, and albumin were compared across tertiles and genders.

Essential data were retrieved from participants who underwent routine health examinations at a tertiary care hospital from January 1, 2021, through July 14, 2023, and were accessed on January 1, 2024. The Institutional Ethics Committee of Kasturba Medical College and Kasturba Hospital, Manipal, approved the study and waived the consent due to the retrospective nature of the study (numbered IEC1: 276/2023). The Declaration of Helsinki was followed when conducting the study.

### Participants

We retrospectively identified the MetS patients following the modified National Cholesterol Education Program Adult Treatment Panel III (NCEP ATP III) guidelines [24]. Participants who were between 20 and 70 years of age were included. Participants were excluded if diagnosed with thyroid disorders, renal disease, pregnancy or lactation, on 25(OH)D supplement, acute inflammatory disease, cancer, anemia, or CVD.

### Data collection

Demographic information, including age, sex, height, weight, blood pressure measurements, and biochemical parameters, was extracted from electronic hospital records. All measurements were performed using validated protocols and calibrated instrumentation.

### Laboratory measurements

Laboratory measurements included total cholesterol (TC), low-density lipoprotein cholesterol (LDL-C), high-density lipoprotein cholesterol (HDL-C), triglycerides, FPG, postprandial plasma glucose (PPG), glycated hemoglobin (HbA1c), albumin, creatinine, and 25(OH)D. Following standardized procedures, biochemical analyses were conducted on a Cobas 8000 analyzer (Roche Diagnostics, Basel, Switzerland). The laboratory maintained internal and external quality control standards, and assays were conducted by laboratory technicians who were unaware of the participants’ clinical information.

### Estimation of Vitamin D and its classification

25(OH)D was determined by quantitative electrochemiluminescence immunoassay (ECLIA) and stratified into three groups. Deficient: < 20 ng/mL, Insufficient: 21 ng/mL to 29 ng/mL, and Normal: ≥ 30 ng/mL [25]. The TyG index was calculated using the formula: Ln [FPG (mg/dL) * Triglyceride (mg/dL)/2] [16,26]. The TyG-body mass index (TyG-BMI) was calculated as the product of TyG and BMI [23].

### Statistical analysis

Data was analyzed using Jamovi (V 2.6.26). Mean ± standard deviation or median (interquartile range) is used for expressing the data. Gender comparisons were conducted using independent samples t-tests or Mann-Whitney U tests for normally and non-normally distributed variables, respectively. Comparisons across TyG index tertiles were performed using One-way ANOVA or the Kruskal-Wallis test for parametric and non-parametric data.

Correlation analysis was performed using Spearman’s rank correlation and Pearson’s correlation coefficient. The two-tailed cutoff point for statistical significance was established at P < .05.

## Results

### General characteristics

This study included 1,297 patients with MetS; 869 (67%) were men, and 428 (33%) were women. For men, median age was 48 [15] years, and 54 [12] for women. In the study population, 767 patients (59%) had VDD, 438 (34%) had insufficient levels, and 92 (7.0%) had normal levels.

### Comparison between tertiles

Table 1 shows the comparison among tertiles. Analysis of TyG index tertiles showed significant differences in lipid profile parameters, including TC, triglycerides, and HDL-C (all P < 0.001). Blood glucose indices, FBG, PPBG, and HbA1c also showed significant differences among tertiles (all P < 0.001).

**Table 1:** Comparison of variables between the tertiles(T).

Variables	T1(n = 425)	T2(n = 437)	T3(n = 435)	P
Age (Years)	51[15]	49[16]	51[14]	0.44
Systolic blood pressure (mmHg)	140[25]	135[24]	138[28]	0.27
Diastolic blood pressure (mmHg)	90[10]	88[10]	90[10]	0.35
Body mass index (Kg/m^2^)	28.4[6.33]	27.7[5]	27.1[5.10]	0.002
Total cholesterol (TC) (mg/dL)	188 ± 40.2	200 ± 41.6	211 ± 44.4	<0.001
LDL-C (mg/dL)	131 ± 35.9	137 ± 38.5	136 ± 39.1	0.06
Vitamin D (ng/mL)	17.8[9.2]	18.9[9.2]	18.3[9.5]	0.49
Deficient (%)	268(63)	241(55)	258(60)	–
Insufficient (%)	128(30)	165(38)	145(33)	–
Normal (%)	29(7)	31(7)	32(7)	–
Fasting blood glucose (mg/dL)	105[13]	109[26]	155[97]	<0.001
Triglyceride (mg/dL)	149[54]	199[54]	264[140]	<0.001
HDL-C(mg/dL)	38[9]	36[9]	36[9]	<0.001
TC/HDL-C	4.9[1.6]	5.4[1.7]	5.7[1.8]	<0.001
HbA1c (%)	5.9[0.8]	6.1[1.3]	7.8[3.4]	<0.001
PPBG (mg/dL)	120[47]	135[72]	230[158]	<0.001
Urea (mg/dL)	21[8]	20[7]	20[9]	0.80
Uric acid (mg/dL)	5.3[1.9]	5.5[1.9]	5.1[2.1]	<0.001
Creatinine (mg/dL)	0.85[0.25]	0.88[0.24]	0.86[0.24]	0.02
Albumin (mg/dL)	4.6[0.3]	4.6[0.3]	4.6[0.4]	0.25
TyG index	8.96[0.28]	9.33[0.19]	9.87[0.49]	<0.001
TyG-BMI	251[54]	259[44.8]	269[52.2]	<0.001

A p-value less than 0.05 indicates statistical significance.

Postprandial blood glucose (PPBG), low-density lipoprotein cholesterol (LDL-C), high-density lipoprotein cholesterol (HDL-C),

Furthermore, we observed statistically significant disparities in kidney function indicators, including creatinine (p = 0.01) and albumin (p < 0.001). Similarly, the TyG-BMI is significantly higher in tertile 3 (p < 0.001).

### Comparisons of gender

Table 2 shows the comparison of genders. Males had significantly higher serum 25(OH)D concentrations than females (P < 0.001). BMI differed significantly between sexes (P < 0.001), with women demonstrating higher BMI values than men. Conversely, men exhibited significantly higher values for the TyG index and triglyceride levels (all P < 0.001) than women. Women participants showed significantly higher values of (HbA1C, TC, and LDL-C (P < 0.001), compared to their male counterparts. TyG-BMI was significantly higher in females compared to males (P = 0.04)

**Table 2:** Sex-specific comparison of clinical and biochemical parameters.

Variables	Male (n = 869)	Female (n = 428)	P
Age (Years)	48[15]	54[12]	<0.001
Body mass index (Kg/m^2^)	27.3[5.10]	28.7[6.33]	<0.001
Systolic blood pressure (mmHg)	136[27]	140[28]	0.06
Diastolic blood pressure (mmHg)	90[10]	84[10]	0.02
LDL-C (mg/dL)	132 ± 36.1	140 ± 40.8	<0.001
Total cholesterol (TC) (mg/dL)	197 ± 41.8	205 ± 45.1	0.002
Vitamin D (ng/mL)	19.2[8.87]	16.6[9.50]	<0.001
Fasting blood glucose (mg/dL)	112[40]	113[46]	0.05
Triglyceride (mg/dL)	199[93]	168[75]	<0.001
TyG index	9.38[0.60]	9.22[0.63]	<0.001
TyG-BMI	258[47]	265[57]	0.04
HDL-C (mg/dL)	35[7]	41[10]	<0.001
TC/HDL-C	5.5[1.7]	5[1.6]	<0.001
HbA1c (%)	6.1[1.8]	6.5[2.1]	<0.001
PPBG (mg/dL)	139[100]	152[118]	0.01
Urea (mg/dL)	21[8]	19[8]	0.02
Creatinine (mg/dL)	0.93[0.17]	0.70[0.15]	<0.001
Albumin (mg/dL)	4.7[0.30]	4.5[0.30]	0.002
Uric acid (mg/dL)	5.8[2.02]	4.6[1.3]	<0.001

A p-value less than 0.05 indicates statistical significance.

Postprandial blood glucose (PPBG), low-density lipoprotein cholesterol (LDL-C), high-density lipoprotein cholesterol (HDL-C),

### Correlation analysis

**Fig 2:**
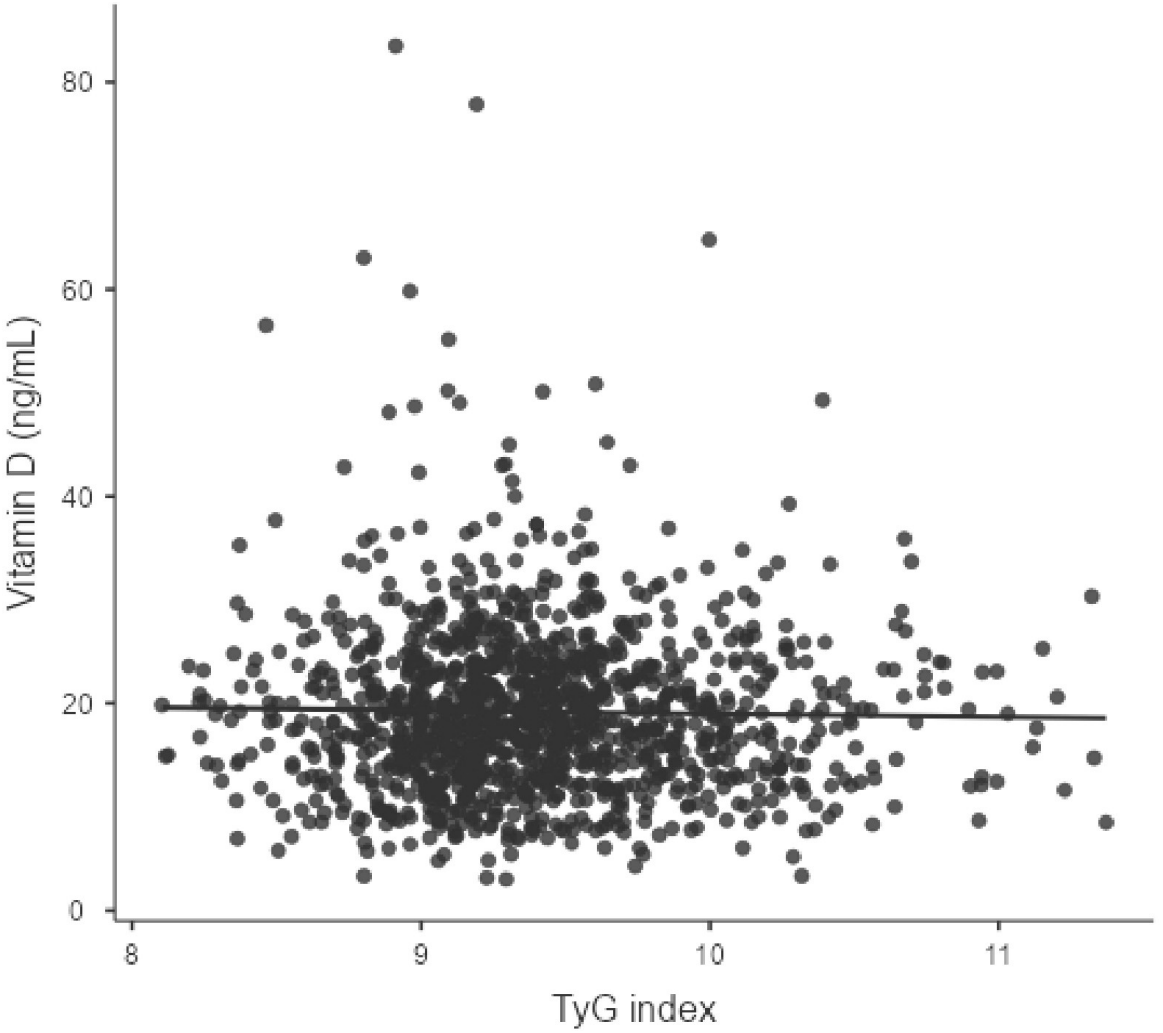
Association between TyG index and serum vitamin D concentration (r = 0.007, p = 0.81): Correlation coefficients were interpreted as follows: r < 0.10 = negligible, 0.10–0.29 = weak, 0.30–0.49 = moderate, ≥ 0.50 = strong association‌‌.

**Fig 3:**
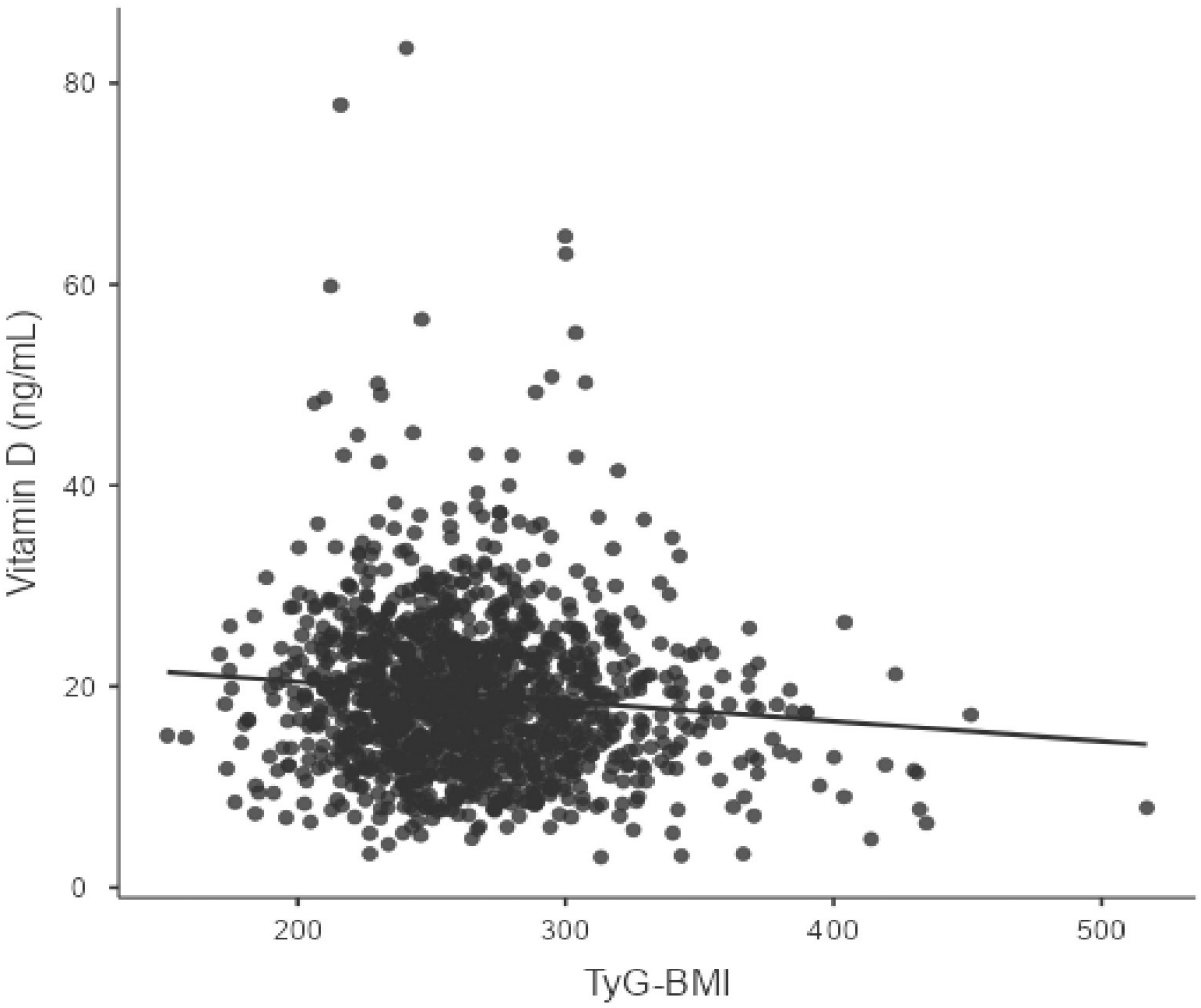
Association between TyG-BMI index and serum vitamin D concentration (r = −0.09, p < 0.001): Correlation coefficients were interpreted as follows: r < 0.10 = negligible, 0.10–0.29 = weak, 0.30–0.49 = moderate, ≥ 0.50 = strong association.

Table 3 depicts the correlation analysis. The TyG index demonstrated significant correlations with lipid profile (TC, HDL-C, and TC/HDL-C) and glucose parameters (HbA1c and PPBG) (all P < 0.001) but not with 25(OH)D. However, TyG-BMI shows a significantly weak negative correlation with vitamin D (r = −0.09, P < 0.001). The scatter plot is shown in (Figs 2 and 3).

**Table 3:** Correlation analysis.

Variables	TyG index		TyG-BMI	
	r	P	r	P
Age (Years)	-0.04	0.12	-0.17	<0.001
Systolic blood pressure (mmHg)	-0.02	0.41	-0.03	0.27
Diastolic blood pressure (mmHg)	-0.03	0.33	0.04	0.17
Body mass index (Kg/m^2^)	-0.12	<0.001	0.92	<0.001
LDL-C (mg/dL)	0.04	0.12	-0.008	0.76
Total cholesterol (TC)(mg/dL)	0.24	<0.001	0.03	0.34
HDL-C (mg/dL)	-0.15	<0.001	-0.05	0.06
TC/HDL-C (mg/dL)	0.34	<0.001	0.08	0.003
HbA1c (%)	0.43	<0.001	0.12	<0.001
PPBG (mg/dL)	0.49	<0.001	0.14	<0.001
Albumin (mg/dL)	0.14	<0.001	-0.15	<0.001
Uric Acid (mg/dL)	-0.07	0.02	0.14	<0.001
Creatinine (mg/dL)	0.02	0.49	-0.07	0.01
Urea (mg/dL)	0.01	0.70	-0.02	0.42
Vitamin D (ng/mL)	0.007	0.81	-0.09	<0.001

A p-value less than 0.05 indicates statistical significance.

Postprandial blood glucose (PPBG), low-density lipoprotein cholesterol (LDL-C), high-density lipoprotein cholesterol (HDL-C); Correlation coefficients were interpreted as follows: r < 0.10 = negligible, 0.10–0.29 = weak, 0.30–0.49 = moderate, ≥ 0.50 = strong association.

## Discussion

This study examined the relationship between 25(OH)D status and TyG-related indices in adults with MetS, revealing limited or no utility for identifying vitamin D deficiency in adults with MetS. Our primary finding is that, while the TyG-BMI showed a statistically significant but weak negative correlation with serum 25(OH)D levels (r = −0.09, P < 0.001), this association is unlikely to be clinically relevant. The TyG index showed no significant association (r = 0.007, P = 0.81). Importantly, this study revealed a high prevalence of VDD and insufficiency in the MetS patients, with only 7% of participants exhibiting normal levels. Significant differences were observed across TyG index tertiles in various metabolic parameters, including lipid profiles, glycemic indices, and markers of kidney function.

Contrary to previous studies suggesting improvements in insulin responsiveness with the dietary addition of 25(OH)D [10,11], the TyG index doesn’t show a significant association with circulating 25(OH)D in our sample; however, the TyG-BMI and 25(OH)D association is consistent with these findings. The very small effect size limits the clinical utility of TyG-BMI as a marker of vitamin D status. Our finding on the TyG index aligns with systematic reviews and meta-analyses of obese and diabetic patients from Brazil, Europe, and the United States, which reported no improvement in insulin resistance after consuming 25(OH)D [12]. Further, a study of Kenyan patients with T2DM also shows no association of insulin resistance with inadequate serum 25(OH)D [27].

The observed widespread VDD underscores the urgent need for a targeted approach to raise 25(OH)D status in this high-risk population. These findings align with previous research documenting a lack of sufficient 25(OH)D among general adults in the Delhi region [3] and MetS patients in northern India [28]. However, the prevalence rate documented in our study exceeded that described in meta-analyses of general adult populations in South Asia [4] and African regions [29]. This inconsistency could be associated with factors such as insulin resistance, a key component of MetS, which has been inversely related to 25(OH)D supplementation [10]. Moreover, MetS patients are usually obese, and it was found that inadequate serum 25(OH)D is linked with higher BMI [10]. Furthermore, a meta-analysis of overweight and obese adults revealed that a lack of 25(OH)D is related to dyslipidemia [30].

The significant variations in lipid profiles across TyG index tertiles are consistent with previous research demonstrating the strong correlation of the index with dyslipidemia [31]. The observed differences in FBG, PPBG, and HbA1c across TyG index tertiles highlight the possible role of the index in identifying individuals with impaired glucose metabolism, consistent with the Simental-Mendía et al. study [16].

Significant differences in kidney function parameters, particularly creatinine and albumin, were observed across TyG index tertiles. These findings indicate a possible connection between insulin resistance, as captured by the TyG index, and initial signs of kidney dysfunction. This association aligns with recent studies reporting correlations between chronic kidney disease (CKD) risk [32] and renal impairment in adults with elevated blood pressure [21] with the TyG index. The underlying mechanisms may involve insulin resistance-mediated pathways, including oxidative stress, inflammation, and endothelial dysfunction [32,33]. However, as the present study is a cross-sectional study, causal inference cannot be established, and the above mechanistic interpretations are purely speculative and require confirmation through longitudinal and interventional studies.

Further, the inverse relationship of the TyG index with BMI contradicts previous findings [34], challenging the conventional understanding of metabolic risk factors. These results imply that the TyG index might capture aspects of metabolic dysfunction independent of, or differently related to, overall adiposity, highlighting the potential limitations of relying solely on BMI as a metabolic risk indicator [35].

The subgroup analysis revealed that women in our study were generally older and had higher BMI than men, consistent with previous research suggesting increased risk for MetS in postmenopausal women [36]. Although the degree of VDD was comparable in both sexes, serum concentrations of 25(OH)D were much higher in men. This finding diverges from a study of adult populations across Latin American countries, which revealed a greater rate of reduced 25(OH)D concentration in men [37]. The decreased 25(OH)D concentrations in women may be attributed to cultural factors in the region, including increased indoor activity, clothing practices that limit sun exposure, and generally higher body fat percentage than men [4,5].

The strengths of this study include its large sample size (n = 1,297) of MetS patients, allowing for robust statistical analysis of various metabolic parameters and sex-specific comparisons. Nonetheless, this study possesses certain drawbacks. Firstly, the cross-sectional approach hinders the capability to determine causal connections. Research spanning an extended period is necessary to evaluate the ability of TyG and TyG-BMI to predict metabolic outcomes and determine whether the temporal variation in the TyG index correlates with changes in the observed parameters. Second, the lack of multivariable adjustment for key confounders such as age, BMI, physical activity, sun exposure, and seasonal variations represents a critical limitation that may substantially affect the observed associations. Furthermore, it was not determined whether subjects were taking medications commonly prescribed for MetS, such as statins or metformin, which are known to influence both lipid profiles and vitamin D metabolism. Future studies should incorporate multivariable-adjusted models to better isolate the independent contribution of TyG-related indices to vitamin D status. Third, this study was conducted on patients with MetS from South India; this may restrict the applicability of our findings to populations from other regions and individuals with different health conditions. Finally, we assessed serum 25-hydroxyvitamin D concentrations, which, while widely accepted as reliable markers of 25(OH)D status, may not fully represent the biochemically active form, 1,25-dihydroxyvitamin D. Furthermore, sensitivity analysis was not conducted in this study. This potential discrepancy could introduce information bias into our results.

## Conclusion

This analysis reveals the high prevalence of VDD and insufficiency in MetS. Moreover, given its significant associations with these clinical parameters, the TyG index emerges as a promising biomarker for assessing lipid metabolism disorders, glycemic control, and renal dysfunction in this population. However, the clinically insignificant association between TyG indices and serum 25(OH)D suggests limited utility for identifying VDD risk within MetS patients.

Future research should also address the identified limitations by incorporating data on unmeasured confounders, such as dietary intake, medication use, and detailed sun exposure habits. Further mechanistic studies are needed to elucidate the complex interplay between insulin resistance, 25(OH)D metabolism, and the progression of MetS components. Public health authorities should prioritize proper screening programs and targeted interventions to address the high rate of VDD in MetS patients, especially considering regional and cultural factors.
